# Supplementary material for: Effect of partner support on antenatal care visits among married adolescents in rural northwestern Uganda: A quasi-experimental study
Source: PLoS One. 2026 Apr 2;21(4):e0346040. doi: 10.1371/journal.pone.0346040 (PMC13046159; doi:10.1371/journal.pone.0346040)
Supplement: S 2 File — (DOCX) [file pone.0346040.s002.docx]

# **** Stata v 15.0 statistical codes ****

** Variables: Assume the outcome variable is denoted by "Y", the exposure is denoted by "Treat", and the covariates are denoted as X1, X2, X3, X4, and X5.

** Assume the dataset is already imported in Stata v15.0.

** Complete Stata do file for conducting the effect of partner support on the frequency of ANC **visits as well as ≥4 ANC visits among married adolescents in rural Uganda

# ** Installing ado-files if using Stata v15.0 or if not already installed

// Downloaded by entering the following command in stata [internet connection needed]:

net from http://personalpages.manchester.ac.uk/staff/mark.lunt

// then clicking on “propensity” and finally clicking on “click here to install”. We also used the //pbalchk command which can be installed in the same way

# ** Propensity score weighting using inverse probability of treatment weighting

*. Uses inverse probability weights to create a pseudo-population.

* Step 1: Check balance before weighting

pbalchk Treat X1 X2 X3 X4 X5

* Step 2: Estimate propensity scores using logistic regression

logistic Treat X1 X2 X3 X4 X5

*Step 3: Predict propensity scores

predict propensity

*Step 4: Diagnosis of propensity scores

estat gof, group(10) table

*Step 5: Use the propensity scores to weight the data.

propwt Treat propensity, ipt smr

// This will create two new kind of variables (weights) in the data, standardized mortality/morbidity // ratio weights (smr_wt) and inverse probability of treatment (ipt_wt)

* Step 6: Recheck balance after weighting

pbalchk Treat X1 X2 X3 X4 X5, wt(ipt_wt) // using ipt_wt weights

*Step 7: Run weighted poisson model and report risk ratios

poisson Y Treat [pw=ipt_wt], irr

*Step 7: Run weighted modified poisson regression after creating a binary outcome Y* from Y

poisson Y* Treat [pw=ipt_wt], irr

# **. Additional analysis for Poisson regression model

**. Assessing the effect of treatment Naively

* Step 1: Perform crude poisson regression

poisson Y Treat, irr

* Step 2: Perform adjusted poisson regression

poisson Y Treat X1 X2 X3 X4 X5, irr

# **. Additional analysis for modified Poisson regression model

* Step 1: Perform crude modified poisson regression

poisson Y* Treat, irr

*Step 2: Perform adjusted modified poisson regression

poisson Y* Treat X1 X2 X3 X4 X5, irr
