# Supplementary material for: Effect of partner support on antenatal care visits among married adolescents in rural northwestern Uganda: A quasi-experimental study
Source: PLoS One. 2026 Apr 2;21(4):e0346040. doi: 10.1371/journal.pone.0346040 (PMC13046159; doi:10.1371/journal.pone.0346040)
Supplement: Attachment — Submitted filename: Response to Reviewers.docx [file pone.0346040.s005.docx]

**Response to Reviewer Comments**

**Dear Editor-in-Chief,**

We thank you and the reviewer for the valuable comments which have helped to improve the quality of the manuscript. We have addressed all the reviewer comments and indicated in the response letter how each comment was addressed by replicating the text in the manuscript.

**Response to Reviewer #1:**

Reviewer #1: Overall
This is a relevant article based on thorough analyses of data.

**Introduction**
Comment: Introduction doesn't always flow well. It can use some improvement. The sentence starting in line 57 does flow well, specifically the part "including the role of the partner [7],".

**Response: We appreciate this comment. We have made some improvements in the introduction section to ensure easy flow. In page 3 (line 52-54), we have added the following text: “Global estimates indicate that 295,000 women died of pregnancy and childbirth-related complications in 2017. Moreover, there are 42 births for every 1,000 teenage girls, with the majority of them being in sub-Saharan Africa (SSA)”**

Comment: The sentence starting in line 62 needs the addition of "from the partner/husband" after the following. "By the partner" at the end of the sentence can then be removed.

**Response: The text "from the partner/husband" has been added, and the text "By the partner" has been removed.**

Comment: The word "Moreover" at the beginning of the sentence starting at line 64 can be removed.

**Response: We have removed the word “Moreover” in line 64. The revised text now reads as follows: “**

**Male participation in ANC is linked to improved access to skilled birth attendance, birth preparedness and complication readiness, including fewer delays in seeking medical attention when needed”.**

Comment: In the paragraph starting at line 76, the inadequacy of cross-sectional study designs is discussed. This remark sounds odd, given that the current study is also using cross-sectional data. You are grading down your study, before it has even started. Later on it becomes clear that the data analysis technique corrects for this, but perhaps other studies with a cross-sectional have done the same. Then it is suggested that the cross-sectional design is not the restriction in this study.

**Response: We agree with this comment. We have removed the following downgrading text: “Since the previous studies used a cross-sectional design, an approach that precludes rigorous approaches to address confounding and selection bias between adolescents with and without partner support.”**

**Method**
Comment: In line 95 it is mentioned that there are 1,172 teenage pregnancies per annum. To judge for themselves if this a high prevalence, the reader needs to know among how many pregnancies in total this is.

**Response: Thank you and we agree with your comment. To address these concerns, on page 4, we have revised the text, and it now reads as follows:**

**“According to the 2024 national population census, Arua City has an estimated 26,770 adolescent girls aged 10 to 19 years. Data from the District Health Information Management System, version 2 (DHIMS2), indicate that 1,172 of these adolescents became pregnant and sought ANC services in health facilities across Ayivu West during the first and second quarters of the 2023/2024 financial year.”**

Comment: In line 99, add participants after 409.

**Response: We are grateful for this observation. We have added the word “participants” after “409”. The revised text on page 4 now reads as follows:**

**“The parent study used a sample size of 409 participants, estimated using William Cochran’s formula.”**

Comment: How were the participants approached? What kind of relationship was there between the data collectors and the participants?

**Response: We thank you for this comment. On page 4 in the “Materials and methods” section, we have added text to address these concerns. The text reads as follows:**

**“During data collection, participants were provided with a detailed explanation of the study objectives, procedures, potential benefits, risks, and their right to voluntary participation, including the freedom to withdraw at any stage without consequences. Recognizing the potential vulnerability of adolescents and the sensitive nature of the study topic, research assistants first established trust by assuring participants of strict confidentiality and privacy throughout the research process. This approach fostered open and honest discussions, allowing participants to express themselves freely without fear of judgment or disclosure of personal information. Additionally, all research assistants signed confidentiality agreements to formally commit to maintaining the privacy and confidentiality of all collected data.”**

Comment: Line 149 says 19 to 14, I assume this the 19 must be 10.

**Response: Again, we are grateful for this observation. On page 6, under the sub-heading 'covariates', the text in part reads as follows:**

**"We included the following baseline covariates: age as a categorical variable (10 -14 versus 15-19 years),.........."**

Comment: Line 152: the cut-off for parity is 3 children. What is the rational for this? The reader will be interested in knowing what it is like, with a first child compare to more children.

**Response: We thank you again for this observation. The cut-off for parity was not 3 children. There was a mismatch between the categories of parity in line 159 and Table 1. We have corrected this anomaly and in line 152, the categories of parity are now (1 to 2 versus 3 to 4) in line with the ones in Table 1. This categorization signifies mothers with less vs. more experience with ANC, delivery, and skilled birth attendance, and postnatal care. We have now provided a rationale for the categorization of parity and of travel time. On page 6, the text reads as follows:**

**“The categorization of parity signifies mothers with limited versus greater experience in utilizing maternal health services, including ANC, skilled birth attendance, and postnatal care. Categorizing travel time as <1 hour, 1-2 hours, and ≥3 hours enables assessment of how increasing distance affects ANC use.”**

Comment: Line 153 indicates time to reach a health facility for ANC. It is more logical to use <1 hour, 1-2 hours, 3-4 hours and > 4 hours.

**Response: We agree with this comment that it is logical to have more categories of time to reach a health facility. However, the level of measurements is the default option (3 categories only) in the parent study, so we could not change them as suggested. In the “study strengths and limitations” section, we have added a text to address these concerns on page 15, and it reads:**

**“Some categorical variables in the previous study may not have been optimally categorized, and reclassification was not possible due to data limitations. For instance, travel time could have been more appropriately categorized as <1 hour, 1–2 hours, 3–4 hours, and >4 hours to better capture differences in access to health facilities.”**

**Results**
Comment: Line 194 says "A higher proportion of Catholic adolescents had supportive partners (76.6%) compared to their Muslim (6.8%) and Pentecostal/Adventist (8.3%) counterparts." This is logical as Catholics are by far the largest group. What is of more interest to the reader is that among the Catholics 76.2% had a supportive partner, among Muslims 66.6.% and among Pentecostal 50%. (Of course even then these are unadjusted findings.)

**Response: We appreciate this comment. We have re-worked the text on page 8 to be of interest to the readers as suggested. The text now reads “Among the Catholics, 76.6% had a supportive partner, among Muslims, 6.8% and among Pentecostal/Adventists, 8.3%”**

Comment: Line 199 mentions "Furthermore, 66.8% of adolescents with partner support demonstrated adequate ANC utilization." How is this for adolescents without partner support?

**Response: We appreciate this comment. Concerning adolescents without partner support, we have added “compared with 51.3% of adolescents without partner support” at the end of the sentence. On page 8, the revised text now reads:**

**“Furthermore, 66.8% of adolescents with partner support demonstrated adequate ANC utilization compared with 51.3% of adolescents without partner support”.**

Comment: Table 2: the numbers for yes and no in partner support are switched around.

**Response: We are grateful for this observation. We have corrected this typing error in Table 2. The number are now accurately reflected in Table 2.**

Comment: Line 237: the term "causal analysis" is used here for the first time. The term is not, but should be, explained in the method section.

**Response: We thank you. In the “Materials and methods” section, the statistical analysis sub-section. We included the text, “examine whether partner participation directly influences ANC utilization”, to explain the meaning of causal analysis. On page 7, the revised text fully reads:**

**“After achieving covariate balance, to examine whether partner participation directly influences ANC utilization (causal analysis), a propensity-score weighted Poisson regression model, adjusted for propensity score weights, was employed to examine the causal effect of partner support on the primary outcome.”**

**Discussion**
Comment: Line 267. The word "explain" is not adequate here. It needs to be replaced by "... may have contributed to men giving partner support." This is what you are exploring here.

**Response: We thank you for this suggestion. The word “explain” has been removed and replaced by the suggested text. The text on page 14 in part now reads “Several factors may have contributed to men giving partner support.”**

Comment: Line 274: I miss reference to articles that already explored this and suggestions for further research how this can be done.

**Response: We have now included on page 18 appropriate references that explored male participation in ANC utilization. They included references 37 and 38 here:**

- **Fletcher R, Forbes F, Dadi AF, Kassa GM, Regan C, Galle A, et al. Effect of male partners' involvement and support on reproductive, maternal and child health and well‐being in East Africa: A scoping review. Health Science Reports. 2024;7(8). doi: 10.1002/hsr2.2269.**
- **Suandi D, Williams P, Bhattacharya S. Does involving male partners in antenatal care improve healthcare utilisation? Systematic review and meta-analysis of the published literature from low- and middle-income countries. Int Health. 2020;12(5):484-98. doi: 10.1093/inthealth/ihz073. PubMed PMID: 31613327; PubMed Central PMCID: PMCPMC11701106.**

**Suggestion for further research was highlighted in the conclusion section as “Future investigation is needed to explore barriers to partner support in promoting maternal healthcare utilization”.**

Comment: Line 282: "hold" should be replaced by "... most likely will hold...".

**Response: Thank you for the suggestion. The word “hold” has been replaced by “most likely will hold”**

Comment: Line 292: the end of the sentence needs adding something like "...and possibly influence the results." That is why you can only say most likely will hold...

**Response: We are grateful for this comment. We have added the suggested text at the end of the sentence. The text in part reads:**

**“While adjustments were made for confounding variables using propensity-score weighting, unmeasured confounders may not have been completely removed and possibly influence the results”.**
