# Supplementary material for: Effect of partner support on antenatal care visits among married adolescents in rural northwestern Uganda: A quasi-experimental study
Source: PLoS One. 2026 Apr 2;21(4):e0346040. doi: 10.1371/journal.pone.0346040 (PMC13046159; doi:10.1371/journal.pone.0346040)
Supplement: Attachment — Submitted filename: Review_PLOS ONE.docx [file pone.0346040.s004.docx]

**Title:** Effect of partner support on antenatal care visits among married adolescents in rural northwestern Uganda: a quasi-experimental study

**General Comment:** The manuscript is relevant to the journal and public health issues in general. It tackles the issue of teenage pregnancy and uses robust statistical approach to measure the impact of partner support on ANC attendance. It is generally a well written, logical, and coherent manuscript.

However, there are some issues that need addressing.

1) There are some statistics presented in the methods, for example “approximately 1172 pregnancies per annum”, estimated population of “137606” in West Nile, among others where the sources of these statistics are not stated. Also, is 137606 the population of Nest Nile or not? There is a need to be specific: Is it for the study sub-counties, Arua as a district, West Nile as a sub-region, and is it for only adolescents? Please clarify
2) What is your decision behind using the Poisson regression model? Did you check the equality of mean and variance? What was the distribution of your primary outcome? You need to explain your choice of this model better. Otherwise, there are other alternative to Poisson model.
3) Also, the prevalence of 4 or more ANCs was 62.6% (very high). Don’t you think might have overestimated the odds ratios? Again, explain the reason behind choosing the model over its alternatives.
4) In Table 2, correct the mistake in column 2, where you state: All (n = 28). I think you meant n = 281
5) In table 3, you define SMD as the absolute standardized mean difference, yet SMDs are presented in mix of positives and negatives. Please check
6) You state that exposure (partner support) preceded the outcome (ANC). How are you sure about that? Did you ask the respondent about it? Some of these adolescents get pregnant when they are in schools, living with their parents, and are later forced to go and live with their husbands. How was the study sure that they started attending ANC, having the support of their husbands?
7) One limitation I think should be added is the sample size issue. It is indeed very small: 281 adolescents. Is this generalizable?
