# Supplementary material for: Effect of partner support on antenatal care visits among married adolescents in rural northwestern Uganda: A quasi-experimental study
Source: PLoS One. 2026 Apr 2;21(4):e0346040. doi: 10.1371/journal.pone.0346040 (PMC13046159; doi:10.1371/journal.pone.0346040)
Supplement: Attachment — Submitted filename: Response_to_Reviewers_auresp_2.docx [file pone.0346040.s006.docx]

**Response to Reviewer Comments**

**Dear Editor-in-Chief,**

We sincerely thank you and the reviewers for the constructive and insightful comments which have helped to improve the quality of the manuscript. We have addressed all the reviewer comments and indicated in the response letter how each comment was addressed by replicating the text in the manuscript.

**Reviewer #1: (No Response)**

**Response to Reviewer #2:**

Reviewer #2: Effect of partner support on antenatal care visits among married adolescents in rural north-western Uganda: a quasi-experimental study

**Abstract**

Comment 1: Why are the authors reporting RR and OR?

**Response: Thank you for this comment. The choice of the measure of effect was guided by the nature of the outcome variables and the statistical models employed. For the primary outcome (number of ANC visits), which is a count variable, we used a Poisson regression model. The natural effect measure from Poisson regression is the risk ratio (RR), which we believe is appropriate. For the secondary outcome (attendance of ≥4 ANC visits), which is a binary outcome, we have now employed the modified Poisson regression, yielding risk ratios (RRs) directly in the revised manuscript.**

Comment 2: Just the at least 4 ANC visits may not really determine the women received the quality services. I suggest that the authors read these two papers to relook and/or refocus their analysis: <https://journals.plos.org/plosone/article?id=10.1371/journal.pone.0263650> and <https://mjz.co.zm/index.php/mjz/article/view/775>

**Response: We appreciate this comment and agree that ≥4 ANC visits alone may not determine the quality ANC utilization, particularly when visits are not timely or when not all the service components are provided at each visit. One of the recommended papers (the other link is inactive/not working) highlight the risk of over-estimation when ANC visits are aggregated without considering timing and service quality. Unfortunately, the existing dataset used in our study did not capture information on timing of ANC initiation (e.g., first-trimester booking). Given this data limitation, we retained ≥4 ANC visits as a proxy indicator, consistent with national guidelines. To address this concern, on page 5, line 146 under the sub-heading 'outcome', we added some text which in part reads as follows: "To reflect the recommendation, we categorized ANC visits as <4 vs. ≥4 to form the secondary outcome as a utilization proxy, not a quality or timeliness indicator".**

**Also, we explicitly acknowledged this in the ‘strengths and limitations’ section. On page 15, the text in part reads as follows “Also, effects should be interpreted cautiously as there may be a possibility of over-estimation of effective ANC utilization since the visits did not consider the timing”, citing one of the two suggested papers.**

**Introduction**

Comment 3: Line 84: A lack of partner support may result in non-utilization… consider changing this from non-utilisation to under-utilisation to imply general lower uptake than expected.
**Response: We agree with this suggestion. The phrase has been revised from “non-utilization” to “under-utilization” to more accurately reflect reduced uptake relative to recommended ANC standards rather than complete absence of care.**

Comment 4: Start with study design then describe the data sources and variables then analysis then the ethical considerations

**Response: Thank you for this structural suggestion. The ‘Materials and methods’ section has been reorganized to improve logical flow and readability, now presented in the following order: 1). Study design; 2). Data source and study setting; 3). Variables and measurements; 4). Statistical analysis; and 5). Ethical considerations.**

Comment 5: For the outcome variables, consider reviewing them in line with the two papers provided above so that issues of quality and reduction of over-estimation of ANC utilisation is curbed.
**Response: As noted in response to Comment 2, we carefully revisited the outcome definitions. While constrained by the data analyzed, we have clarified that ≥4 ANC visits reflect service contact, not quality or timeliness, and we have included this concern as a study limitation noting. Please see our response to comment #2 above.**

Comment 6: Diagnosis for propensity score and Poisson regression model is more of the methods and should be reported under methods.

**Response: We agree with your suggestion. The subsection on ‘diagnostics for the propensity score and Poisson regression models’ has been moved from the ‘Results’ section to the ‘Statistical analysis subsection’ under ‘Materials and Methods’ section. The Results section now focuses strictly on the study findings.**

Comment 7: Table 4 should include the other variables as well. The variables that you did the adjustment for the effect that you are presenting.

**Response: Thank you for this comment. In causal analyses using propensity score weighting, all relevant covariates are incorporated into the estimation of a single summary measure—the propensity score—which is then used to weight the groups being compared. Consequently, the outcome regression model is adjusted for the propensity score weights rather than for individual covariates. For transparency, we have already listed all covariates used to generate the propensity score as a footnote to Table 4. Please see the footnote under Table 4 on page 13.**

Comment 8: All data are presented in tables, consider using graphs like forest plot to show the uptake of timely ANC visits by the socio-demographic characteristics?
**Response: We thank you for this helpful suggestion. While graphical displays such as forest plots can be useful for visualising effect estimates and confidence intervals, we chose to retain tabular presentation to ensure clarity and consistency with the causal analytic approach used in this study. Specifically, the estimates presented are derived from propensity score–weighted models, for which tables allow more transparent reporting of point estimates, confidence intervals, and model specifications. We therefore retained tables as the primary mode of presentation but acknowledge that graphical displays may complement tabular results in future work.**

Comment 9: May be another forest plot for the adjusted OR for the final model.
Discussion

**Response: Please refer to our response to comment #8 above.**

Comment 10: Discussion has not been reviewed since the definition of the 4 ANC visits should be timely and not just at least 4 visits based on the two papers that have found over-estimation if the visits are lumped.

**Response: Thank you for this comment. In the study, ANC utilization was operationalized as the number of visits (<4 versus ≥4), consistent with the definition available in the secondary dataset used for the analysis. We acknowledge that this measure captures the quantity of ANC visits rather than their timing, and therefore may overestimate effective or timely ANC utilizations, as highlighted in previous studies. We have revised the Discussion to explicitly acknowledge this limitation, clarify that our findings relate to attendance of at least four ANC visits rather than timely ANC attendance, and caution against interpreting the results as reflecting adequacy of ANC timing. On page 15, we added the following text as a limitation:**

**"ANC attendance was defined as fewer than four versus at least four visits based on the available data. This measure captures the number of ANC contacts but not their timing, which may overestimate timely or adequate ANC utilisation. Therefore, our findings should be interpreted as reflecting attendance of at least four ANC visits rather than adherence to recommended timely ANC schedules. Future studies with detailed information on visit timing are needed to better align measurement with current WHO recommendations."**

**Response to Reviewer #3:**

Reviewer #3: Effect of partner support on antenatal care visits among married adolescents in rural northwestern Uganda: a quasi-experimental study

**General Comment:** The manuscript is relevant to the journal and public health issues in general. It tackles the issue of teenage pregnancy and uses robust statistical approach to measure the impact of partner support on ANC attendance. It is generally a well written, logical, and coherent manuscript. However, there are some issues that need addressing.

**Response: Thank you for the encouraging comment.**

Comment 1: There are some statistics presented in the methods, for example “approximately 1172 pregnancies per annum”, estimated population of “137606” in West Nile, among others where the sources of these statistics are not stated. Also, is 137606 the population of Nest Nile or not? There is a need to be specific: Is it for the study sub-counties, Arua as a district, West Nile as a sub-region, and is it for only adolescents? Please clarify

**Response: We appreciate this observation and agree that clarity and proper sourcing of contextual statistics are essential. We have explicitly cited the sources of the statistics. On page 4, we clarified the geographic and demographic scope of each statistic: For example, 3,316,255 instead of 137,606 refers to the total population of the West Nile sub-region, not adolescents only. The reworked text on page 4, line 108 reads as follows:**

**“****The West Nile region borders the Democratic Republic of Congo (DRC) to the west and had an estimated population of 3,316,255 in 2024. Arua City was estimated to have 384,656 residents, of whom 26,770 were adolescent girls aged 10–19 years. During the first and second quarters of the 2023/2024 financial year, 1,172 adolescents became pregnant and sought ANC services across health facilities in Ayivu West.”**

Comment 2: What is your decision behind using the Poisson regression model? Did you check the equality of mean and variance? What was the distribution of your primary outcome? You need to explain your choice of this model better. Otherwise, there are other alternative to Poisson model.

**Response: Thank you for this comment. The primary outcome was the number of ANC visits, which is a count variable with non-negative integer values. The distribution of ANC visits was right-skewed and when we assessed the mean–variance relationship, we found no substantial overdispersion. Also, formal Poisson goodness-of-fit tests using deviance and Pearson statistics indicated that the model fit was adequate (p > 0.90). To ensure clarity in the “Statistical analysis subsection” section on page 7 (line 203), we have added the following text to address the comment:**

**“****We assessed the Poisson regression assumption of equidispersion using the Pearson-based dispersion statistic. Over-dispersion is suggested when the dispersion statistic exceeds one. In our analysis, the Pearson dispersion statistic (Pearson χ²/df = 0.582) was well below this threshold, indicating no evidence of substantial over-dispersion in the data.”**

Comment 3: Also, the prevalence of 4 or more ANCs was 62.6% (very high). Don’t you think might have overestimated the odds ratios? Again, explain the reason behind choosing the model over its alternatives

**Response: We appreciate this comment and acknowledge that when the outcome is common (prevalence >10%), odds ratios (ORs) may overestimate risk ratios (RRs). In the revised manuscript, we have now employed the modified Poisson regression, yielding risk ratios (RRs) directly for our secondary outcome.**

Comment 4: In Table 2, correct the mistake in column 2, where you state: All (n = 28). I think you meant n = 281

**Response: Thank you for identifying this typographical error. The column header in Table 2 has been corrected from All (n = 28) to All (n = 281).**

Comment 5: In table 3, you define SMD as the absolute standardized mean difference, yet SMDs are presented in mix of positives and negatives. Please check

**Response: We appreciate this observation. The negative or positive sign of the absolute standardized mean difference (SMD) simply indicates the direction of imbalance, either on the left or right of the distribution. Therefore, the values are correct.**

Comment 6: You state that exposure (partner support) preceded the outcome (ANC). How are you sure about that? Did you ask the respondent about it? Some of these adolescents get pregnant when they are in schools, living with their parents, and are later forced to go and live with their husbands. How was the study sure that they started attending ANC, having the support of their husbands?

**Response: We thank you for this comment. In this study, partner support was measured with reference to the pregnancy period, specifically support received during ANC attendance. All adolescents included were married at the time of data collection and had reached at least 36 weeks of gestation. Nonetheless, we acknowledge that the cross-sectional design of the parent study does not allow for perfect verification of temporal sequencing, and some adolescents may have initiated ANC before cohabiting with or receiving support from their husbands. To address this, we have added a statement in the ‘Strengths and Limitations’ section (page 15, line 304) to acknowledge potential ambiguity in exposure timing:**

**“The study relied on self-reported data regarding partner support, which may be subject to response bias, including potential ambiguity in the timing of exposure relative to ANC attendance.”**

Comment 7: One limitation I think should be added is the sample size issue. It is indeed very small: 281 adolescents. Is this generalizable?

**Response: We acknowledge that the relatively small sample size limits the generalizability of our findings beyond similar rural settings and married adolescents. In the ‘Strengths and Limitations’ section (page 15, line 302), we added the following text:**

**“****The relatively small sample size supported causal estimation within the study population but limits the generalizability of the findings to other settings or populations with different characteristics.”**

**Response to Reviewer #4:**

Reviewer #4: I am concerned about the selection of adolescents that were married. Is it not likely that adolescents that were married are likely to be the ones accepted by their spouses than those that were not married, Therefore, the married ones were also more likely to receive spousal support. This should be discussed and highlighted as a limitation. Therefore, the final conclusions should be made in light of this limitation.

**Response: The study focused on married adolescents for conceptual and methodological reasons. Partner support, as measured in this study (financial, emotional, accompaniment, and encouragement), presupposes a socially recognized partner. Among unmarried adolescents, partner availability, acknowledgment of pregnancy, and involvement are often inconsistent or absent, making exposure classification unreliable. Including unmarried adolescents could therefore have led to substantial misclassification, as a lack of support may reflect partner absence rather than unwillingness to support.**

**We acknowledge, however, that restricting the sample to married adolescents limits the generalizability of our findings to all adolescents and may result in higher observed levels of partner support than in the broader adolescent population. As such, conclusions regarding partner support should be interpreted within the context of married adolescents, as clearly stated in the revised ‘Strengths and Limitations’ section (page 15).**

**"****This study was restricted to married adolescents, which allowed for reliable measurement of partner support but limits the generalizability of the findings to all adolescents. Unmarried adolescents may experience different levels or types of partner support, and including them could have introduced misclassification of exposure. Consequently, conclusions regarding partner support should be interpreted within the context of married adolescents."**
